# Evaluation of Tannin Extracts, Leonardite and Tributyrin Supplementation on Diarrhoea Incidence and Gut Microbiota of Weaned Piglets

**DOI:** 10.3390/ani11061693

**Published:** 2021-06-06

**Authors:** Matteo Dell’Anno, Serena Reggi, Valentina Caprarulo, Monika Hejna, Carlo Angelo Sgoifo Rossi, Maria Luisa Callegari, Antonella Baldi, Luciana Rossi

**Affiliations:** 1 Department of Health, Animal Science and Food Safety “Carlo Cantoni” (VESPA), Università Degli Studi di Milano, 26900 Lodi, Italy; serena.reggi@unimi.it (S.R.); monika.hejna@unimi.it (M.H.); carlo.sgoifo@unimi.it (C.A.S.R.); antonella.baldi@unimi.it (A.B.); luciana.rossi@unimi.it (L.R.); 2 Department of Molecular and Translational Medicine (DMMT), Università Degli Studi di Brescia, 25123 Brescia, Italy; valentina.caprarulo@unibs.it; 3 Department for Sustainable Food Process (DiSTAS), Università Cattolica del Sacro Cuore, 29122 Piacenza, Italy; marialuisa.callegari@unicatt.it

**Keywords:** alternatives to antibiotics, antimicrobial resistance, feed additives, functional feed, tannins, leonardite, tributyrin, microbiota, diarrhoea incidence, weaned piglets

## Abstract

**Simple Summary:**

Replacing and reducing antimicrobial treatments in livestock farming has become crucial for animal, human and environmental health due to global concerns regarding antimicrobial resistance. Weaning is a stressful phase in which piglets can develop enteric disorders leading to the massive use of antibiotics. In this scenario, functional nutrition represents one alternative to reducing antimicrobial resistance. The aim of this study was to assess the effects of the dietary administration of the combination of tannin extracts, leonardite and tributyrin supplementation on weaned piglets. After weaning, piglets were divided into two experimental groups: one fed a basal diet (CTRL) and one fed a basal diet supplemented with 0.75% Quebracho and Chestnut tannin extracts, 0.25% leonardite and 0.20% tributyrin (MIX), respectively, for 28 days. Zootechnical performance and diarrhoea incidence were recorded. Microbiological and microbiota analyses were performed on faecal samples, and metabolic profile of blood serum was evaluated. The MIX group revealed a reduced incidence of diarrhoea and improved faecal consistency compared to the CTRL group. After 28 days, MIX revealed an increased lactobacilli:coliform ratio compared to CTRL, and the serum metabolic profile showed lower levels of low-density lipoproteins, suggesting a modulation of the lipid metabolism. The results suggest that the combination of tannin extracts, leonardite and tributyrin could improve animal health, thus reducing diarrhoea occurrence in weaned piglets.

**Abstract:**

The effects of the dietary administration of a combination of Quebracho and Chestnut tannins, leonardite and tributyrin were evaluated in weaned piglets. A total of 168 weaned piglets (Landrace × Large White) were randomly allotted to two experimental groups (6 pens/group, 14 piglets/pen). Animals were fed a basal control diet (CTRL) and a treatment diet (MIX) supplemented with 0.75% tannin extracts, 0.25% leonardite and 0.20% tributyrin for 28 days. Individual body weight and feed intake were recorded weekly. Diarrhoea incidence was recorded by a faecal scoring scale (0–3; considering diarrhoea ≥ 2). At 0 and 28 days, faecal samples were obtained from four piglets/pen for microbiological and chemical analyses of faecal microbiota, which were then assessed by V3-V4 region amplification sequencing. At 28 days, blood from two piglets/pen was sampled to evaluate the serum metabolic profile. After 28 days, a reduction in diarrhoea incidence was observed in the MIX compared to CTRL group (*p* < 0.05). In addition, compared to CTRL, MIX showed a higher lactobacilli:coliform ratio and increased *Prevotella* and *Fibrobacter* genera presence (*p* < 0.01). The serum metabolic profile showed a decreased level of low-density lipoproteins in the treated group (*p* < 0.05). In conclusion, a combination of tannin extract, leonardite and tributyrin could decrease diarrhoea incidence and modulate the gut microbiota.

## 1. Introduction

In livestock, alternatives to antibiotics that are capable of promoting the health status, preventing diseases and reducing medical treatments are needed in order to tackle increasing global antibiotic resistance [1,2]. Although it is not totally clear how antibiotic use in food-producing animals spreads resistant bacteria in humans, replacing antimicrobials is a key aim of European policies [3,4].

This need was particularly highlighted by the removal of zinc oxide licensed as a medicinal product as it is involved in environmental pollution and in the co-selection of antibiotic-resistant bacteria [5,6]. In fact, following the ban on the use of antibiotics as growth promoters, therapeutic antibiotics and zinc oxide have become more widely used [7,8,9] to prevent porcine colibacillosis, improve suboptimal weight gain, and feed efficiency. Alternatives to antibiotics and zinc oxide are also urgently needed to guarantee the profitability of swine farming, particularly during weaning, which involves the largest use of antimicrobials due to the high incidence of gastroenteric disorders and multifactorial post-weaning diarrhoea (PWD) [10,11]. The gastrointestinal tract (GIT) is a highly specialised organ where the dynamic interaction between host cells and the complex environment (mucosal chemical barrier, immune system, and epithelium) impacts on gut health [12]. Gut health is important in the reduction of diseases and the optimal functioning of digestive processes, along with optimal production performance.

The aim of nutrition is no longer simply to satisfy nutritional requirements; it also plays a key role in the health and welfare of humans and animals [13]. The nutritional components of animal feed are thus continually adjusted to optimize the effects on animal health and growth. Functional feed ingredients, which sustain the health status and reduce the risk of pathologies, have thus become fundamental in replacing or reducing antimicrobials in food-producing animals [14,15]. Studies have thus focused on developing nutraceutical alternatives to antibiotics in order to maintain swine health and performance [16]. In addition to their nutritional value, functional feed ingredients contain bioactive compounds and nutricines which exert beneficial activities on the organism (immunomodulatory, antioxidant, anti-inflammatory, antibacterial effects, etc.) with positive impacts also on animal performance and general farm profitability [17,18,19]. 

Tannins have antioxidant, anti-inflammatory and antimicrobial properties, and are used to enhance growth performance, modulate intestinal microbiota, and decrease the incidence of diarrhoea, particularly during the post-weaning period [20] through their antimicrobial and cytomodulatory effects on intestinal cells [21]. Hydrolysable and condensed tannins increase performance and animal gut health, and reduce diarrhoea by directly inhibiting enterotoxigenic *Escherichia coli* bacteria when supplemented in weaned piglets [22]. They disrupt the bacterial wall by releasing hydrogen peroxide from their hydroxyl groups [23]. In addition, tannins may reduce cortisol levels in animals and protect against lipid peroxidation, leading to a decreased levels of plasma malondialdehyde [24].

Leonardite is rich in humic acids, and due to its macrocolloid structure can protect intestinal mucosa by reducing the resorption of toxic metabolites from the residues of harmful substances in feed [25]. Humic acids prevent an excessive loss of water from the gut, which is important in the treatment of diarrhoea, dyspepsia, and acute intoxications [26]. In weaned piglets, leonardite improves animal performance and modulates lipid metabolism by increasing the level of HDL cholesterol in blood serum and suggesting an enhanced defence from stressors through a higher Mg serum level after 40 days of supplementation [27]. Leonardite seems to act by helping ion transport through membranes, protecting intestinal mucosa, enhancing enzymatic activities and promoting nutrient digestion and adsorption, particularly for proteins and minerals [28].

Some specific dietary short chain and medium chain fatty acids play a key role in the intestinal inflammation of pigs, as well as in modulating the intestinal microbial population and in promoting digestion [29]. The supplementation of 0.20% of tributyrin was shown to boost animal performance, lipid metabolism and gut health through increased energy metabolism of enteric bacteria and promoting the richness positively related to animal performance and mucosal immune function [30]. Tributyrin can act as a histone deacetylase inhibitor (HDAC), stimulating muscle growth through satellite cell differentiation in muscular tissue promoting animal performance [31]. In addition, butyrate supplementation may increase villus height and crypt depth in the duodenum [32]. Antibacterial activity related to butyrate was observed through a reduction in intestinal pH and a decrease in harmful bacteria in the caecum [33].

Despite the high number of products studied as alternatives to antimicrobials in commercialised feed for swine farming, few studies have specifically investigated the synergistic or antagonistic effects of possible additive combinations on the health and performance of weaned piglets. Due to encouraging results obtained in our previous studies from the supplementation of single additives, the aim of this study was to assess the possible combined effects of Quebracho and Chestnut tannin extracts, leonardite and tributyrin supplementation on animal health and microbiota modulation in weaned piglets. 

## 2. Materials and Methods

### 2.1. Animals, Housing, Experimental Design and Treatment

The experimental trial was approved by the Animal Welfare Organization of the University of Milan (OPBA authorization no. 09/2020) and performed in accordance with European regulations [34]. The trial was conducted on a commercial farm that was free from pathologies included in the ex-list A of the World Organization for Animal Health (Porcine Reproductive Respiratory Syndrome, atrophic rhinitis, transmissible gastroenteritis, salmonellosis and Aujeszky disease).

The study lasted 28 days, and included 168 weaned piglets (Landrace x Large White; 28 ± 2 days) homogeneous by gender (50% male, 50% female) and weight (7.48 ± 1.16 kg). Piglets were identified by individual ear tags and housed in 12 different pens (14 animals/pen), in standardised environmental conditions (27 °C and 60% relative humidity). 

After an adaptation period of three days with the same basal diet, piglets were allotted to a randomised complete block design in two experimental groups: control group (CTRL: 84 piglets, 6 pens) fed the basal diet (ad libitum), and the treatment group (MIX, 84 piglets, 6 pens) fed the basal diet (ad libitum) supplemented with 0.75% Quebracho and Chestnut tannin extracts (Silvafeed^®^ Nutri P, Silvateam, Mondovì, Italy), 0.25% leonardite (New Feed Team, Lodi, Italy) and 0.20% tributyrin (ACIFIS^®^ Tri-B, New Feed Team, Italy), based on previous studies [20,27,30]. The two experimental isoproteic and isoenergetic diets (Table 1) were balanced using Plurimix System^®^ software v. 2.4 (Fabermatica, Cremona, Italy) in order to meet the nutritional requirements for post-weaned piglets [35] and were provided by Ferraroni S.p.A. (Cremona, Italy). Considering the small inclusion percentage, the additives were premixed with wheat flour to ensure a homogeneous dispersion before being added to the horizontal mixer with the other ingredients, substituting 2% of wheat meal with 2% of the experimental mix (0.80% wheat flour 00, 0.75% tannin extracts, 0.25% leonardite, and 0.20% tributyrin).

### 2.2. Chemical Evaluation of Experimental Diets

The experimental diets were analysed in duplicate in terms of principal nutrients: dry matter (DM), ether extract (EE), crude protein (CP), crude fibre (CF), and ash content. Dry matter (DM) was obtained by drying samples in a forced air oven at 65 °C for 24 h (AOAC method 930.15). CP was determined by the Kjeldahl method (AOAC method 2001.11). EE was determined using ether extraction in the Soxtec system (DM 21/12/1998). CF was determined by the filtering bag technique [36]. Ash content was obtained by incinerating samples in a muffle furnace at 550 °C (AOAC method 942.05).

### 2.3. Zootechnical Performance, Diarrhoea Incidence and Sampling Procedures

Body weight (BW) was individually recorded at days 0 (T0), 7 (T1), 14 (T2), 21 (T3) and 28 (T4). Feed intake was recorded weekly for each pen by measuring the feed refused, considering the pen as the experimental unit. Average daily gain (ADG), average daily feed intake (ADFI) and feed conversion ratio (FCR) were calculated. For the microbiological and microbiota analyses, faecal samples were collected at T0 and at T4 from the rectal ampulla of four piglets per pen randomly selected (24 piglets CTRL, 24 piglets MIX).

Faecal consistency of the four selected piglets was scored on a weekly basis using a four-level scale: 0 = normal consistency (faeces firm and well formed); 1 = soft consistency (faeces soft and formed); 2 = mild diarrhoea (fluid faeces, usually yellowish); 3 = severe diarrhoea (faeces watery and projectile). A faecal consistency score ≤ 1 (0,1) was considered normal, whereas a faecal score >1 (2,3) was defined as diarrhoea. Faecal colour was also evaluated using a three-level scale: 1 = yellowish colour; 2 = greenish colour; 3 = brown colour. A faecal colour ≥ 2 (greenish-brown) was considered normal, while a faecal colour < 2 (yellowish) was considered pathological [11]. 

Blood was sampled from the jugular vein of two randomly selected subjects per pen at T0 and T4, using vacuum tubes without any anticoagulant.

### 2.4. Blood Serum Analysis

Serum samples were obtained by centrifugation, and were analysed using a multiparametric autoanalyzer for clinical chemistry (ILab 650; Instrumentation Laboratory Company, Lexington, MA, USA) at 37 °C. We measured the following concentration of: total protein (g/L), albumin (g/L), globulin (g/L), albumin/globulin (A/G ratio), alanine aminotransferase (ALT-GPT; IU/L), glucose (mmol/L), urea (mmol/L), creatinine (µmol/L), total bilirubin (µmol/L), total cholesterol (mmol/L), triglycerides (mmol/L), high-density lipoprotein (HDL; mmol/L), low-density lipoprotein (LDL; mmol/L), phosphorus (mmol/L), and magnesium (mmol/L).

Serum glucagon and insulin concentrations were also quantified using enzyme-linked immunosorbent assay (ELISA) kits specific for pigs according to the manufacturer’s instructions (Mecordia Inc., Uppsala, Sweden; Cusabio Technology Llc, Houston, TX, USA). Absorbances were measured with a microplate reader at 450 nm (Bio-Rad 680 microplate reader, Bio-Rad Laboratories, Inc., Hercules, CA, USA) and concentrations were calculated according to the respective standard curve using CurveExpert v. 1.4.

### 2.5. Microbiological Analysis of Faecal Samples

The faecal samples were analysed in terms of the total bacteria (Plate Count Agar, PCA), lactobacilli (De Man, Rogosa and Sharpe Agar, MRS) and coliform count (Violet Red Bile Broth Agar, VRBA). Briefly, 1 g of each faecal sample was homogenised with 10 mL of sterile physiological solution and centrifuged (3000 rpm for 10 min) to collect the supernatant. Samples were serially diluted, and microorganisms were enumerated by plate counting after 24 h of semi-anaerobic incubation at 37 °C using the overlay method for MRS and VRBA, and the inclusion method for PCA [37,38,39]. The lactobacilli:coliform ratio was calculated based on plate counting results which were expressed as log10 of colony-forming units per gram of faeces (log_10_ CFU/g).

### 2.6. Nitrogen Content, Apparent Nitrogen Digestibility, Volatile Fatty Acids and pH of Faecal Samples

Faeces collected at 28 days were dried in a forced-air oven and analysed for nitrogen content (AOAC method 930.15; AOAC method 2001.11). Apparent nitrogen digestibility was assessed through the acid insoluble ash (AIA) marker [40] after incinerating feed and faecal samples in a muffle furnace at 550 °C (AOAC method 942.05). Apparent nitrogen digestibility was then calculated using the following equation:

(1) $$Apparent\;Nutrient\;Digestibility\;\left(\%\right)=100\times (1-\frac{marker\;in\;feed}{marker\;in\;feaces}\times \frac{nutrient\;in\;faeces}{nutrient\;in\;feed})$$

The fresh faecal sample pH of T4, diluted in 10 mL of sterile physiological solution and subsequently centrifuged, were measured for the supernatant using a pH meter.

Volatile fatty acid analysis was performed by gas chromatography (GC), and the samples were prepared as follows: 0.5 gof faecal samples were dissolved in 1 mL of distilled water and thoroughly mixed for a few minutes. Following centrifugation (10,000× *g* for 10 min at 10 °C), 0.5 mL of supernatant was added to 250 µL of oxalic acid (0.12 M) and 250 µL of pivalic acid solution (1 g pivalic acid + 50 mL formic acid, filled to 1 L with distilled water). After mixing and centrifugation (10,000× *g* for 10 min at 10 °C), the clear supernatant was transferred into the vial, and injected into the GC. Volatile fatty acids (VFA) were quantified according to Ahmed et al. [41].

### 2.7. Bacterial DNA Extraction, V3-V4 Region Amplification and Sequencing

Faecal samples were collected at 28 days and stored in frozen dry ice until further processing. Bacterial DNAs were extracted starting with 50 mg (fresh weight) of faecal sample using the FastDNA™ SPIN Kit for Soil (MP Biomedicals, Eschwege, Switzerland) according to the manufacturer’s instructions. Extracted DNA was quantified using the Qubit HS dsDNA fluorescence assay (Life Technologies, Carlsbad, CA, USA), whereas the DNA quality check was carried out through agarose gel electrophoresis. DNA was sent to Fasteris SA (Geneva, Switzerland) for sequencing. The amplicons were sequenced by Illumina MiSeq v. 3 in 2 × 300 bp mode. Trimmomatics v. 0.32 (http://www.usadellab.org/cms/index.php?page=trimmomatic, accessed on 26 August 2020) [42] was used to remove the adapter sequence from the reads, and during this process, the filtering was performed. Filtering was performed by SLIDINGWINDOW, a sliding window trimming, which cuts the read tail when four consecutive bases are of low quality. By considering multiple bases, a single poor quality base was thus not the cause of the removal of high quality data later in the read (window size: 4 base, quality: 15). Filtering was also performed by MINLEN which drops the read when it is below a specified length (set at 60 bases). 

Filtered reads were mapped against the SILVA database using Burrows–Wheeler Alignment Tool v. 0.7.5a (http://bio-bwa.sourceforge.net/, accessed on 28 August 2020). SAM tools was used to merge alignments and to compute the number of reads onto each OTU. Sequence files are available in the European Nucleotide Archive (ENA) database under accession number ID PRJEB43937.

### 2.8. Statistical Analysis

The results were analysed using a generalised linear model that assesses the leverage of the effects based on the analysis of variances using JMP 14 Pro^®^ (SAS Inst. Inc., Cary, NC, USA). For animal performance, faecal bacterial counts, the model included the fixed effect of treatments (Trt), the effect of time (Time), and the interaction between treatment and time (Trt × Time). Serum metabolites data were evaluated after performing analysis of covariances (ANCOVA) to adjust the initial variability of serum samples. Data on diarrhoea incidence were assessed using Pearson’s Chi-Squared test. The results from faecal nitrogen content, immunoenzymatic kits, apparent digestibility, volatile fatty acids and faecal pH at T4 were analysed using ANOVA. Pearson correlations were performed. Multiple comparisons between groups were evaluated with Tukey’s Honestly Significant Difference test (Tukey’s HSD). Results were presented as least square means ± standard error (SE). Means were considered different when *p* ≤ 0.05.

All statistical analyses concerning sequences obtained were performed using MicrobiomeAnalyst [43,44], which calculates alpha diversity based on Chao 1, Observed species, Simpson and Shannon metrics. Significant differences in these indices were calculated using a t test ANOVA. Beta diversity across samples was instead calculated using the Bray–Curtis index and PERMANOVA statistical methods. The beta diversity across the microbial community of animals belonging to both dietary groups were visualised using a PCoA plot. The edgeR algorithm with 0.05 adjusted *p*-value cut-off was used to identify significant differences in taxa abundance between the two groups of animal microbiota. The Linear discriminant analysis Effect Size (LEfSe) Sparse Correlations for Compositional data (SparCC) and Random Forest analysis were performed using the same tool. 

GraphPad Prism v. 8 (GraphPad Prism, San Diego, CA, USA) was used to perform the t test and Spearman’s correlation analysis, respectively.

## 3. Results and Discussion

### 3.1. Chemical Evaluation of the Experimental Diets

Nutrient profile of both experimental diets was in line with NRC [35] guidelines fulfilling the nutritional requirements of post-weaning piglets. The inclusion in the diet of 0.75% tannin extracts, 0.25% leonardite and 0.20% tributyrin did not influence the main nutrient profiles of MIX group experimental diet (Table S1).

### 3.2. Zootechnical Performance

Zootechnical performance, in swine farming, besides being the main concern for farmers, is considered as an indirect indicator of intestinal health. Both groups revealed a progressive increase in body weight in line with standard growth curves, ADG and ADFI (Table S2). No detrimental effects of the combination of ingredients were observed. 

The results showed no significant differences in BW, ADG, ADFI, FCR between the experimental groups. The effect on the zootechnical performance could well have been more exacerbated with longer experimental periods [27,30]. In fact, for those additives affecting animal production or performance, long-term efficacy and safety studies are necessary, which correspond to periods of 42 days in post-weaning piglets [45,46]. In addition, the positive effects of functional and antioxidant dietary compounds are more evident when animals have a pathological condition (e.g., experiential infection, intestinal injuries) [47,48], whereas in this study the animals were in good general health throughout the experimental period.

### 3.3. Diarrhoea Incidence 

**Figure 1:**
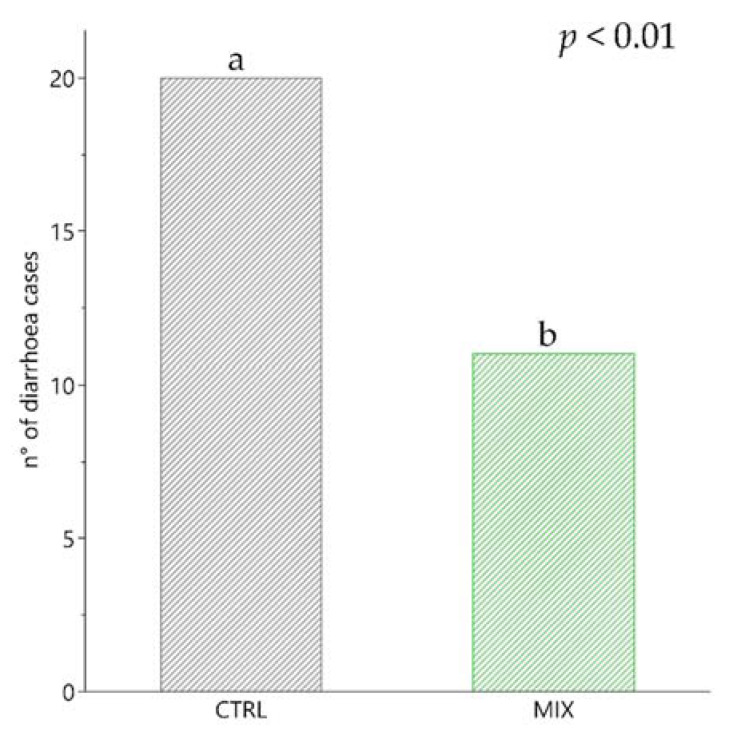
Diarrhoea incidence from 0 to 28 days of experimental period divided per control (CTRL) and treatment group (MIX). ^a^^,^^b^ Means with different superscripts are significantly different between treatments (*p* < 0.05). CTRL: control group; MIX: treatment group supplemented with 0.75% tannin extracts, 0.25% leonardite and 0.20% tributyrin in the diet.

Diarrhoea is one of the main issues in the weaning phase in swine farming and represents the most evident sign of dysbiosis. On the other hand eubiosis, an indicator of gut health, is ensured through a positive interaction between the host, the microorganisms and the environment. At the beginning of the trial, piglets were healthy without any signs of diarrhoea (T0). The highest numbers of diarrhoea were during the first 14 days (17 piglets with faecal score ≥ 2) in both groups. The first two weeks of weaning are considered as the most critical phase for diarrhoea incidence, which is caused by maternal immunity reduction and the impact of stressors, leading to decreased performance and antibiotic use [49]. There was a higher incidence of diarrhoea in the CTRL group (20 cases; 16.67% of faeces evaluated) compared with the MIX group (11 cases; 9.17% of faeces evaluated) throughout the experimental period (*p* < 0.01; Figure 1). Regarding faecal colour, a yellowish colour at 7 days was recorded in only four animals (1 CTRL and 3 MIX) with no statistical differences at any point during experimental period. Post-weaning diarrhoea is recognised as a multifactorial disease. It can be influenced by post-weaning fasting, together with environmental and feeding stress. From a microbiological point of view, many factors could be involved in the aetiology, such as bacteria, parasites and viruses [50]. 

Although the individual effects of each functional component supplemented cannot be differentiated, their combination lowered the occurrence of diarrhoea. Previous results obtained in our studies showed that all three compounds tested separately had a positive effect on gut health. In this study, due to their different mechanisms of action these compounds may also have contributed to the reduction in diarrhoea. In fact, Chestnut and Quebracho tannins are antimicrobial and antioxidant substances with a powerful effect on enterotoxigenic *Escherichia coli,* which are the main pathogens involved in diarrhoea occurrence in weaned piglets [51,52]. Tannins may directly affect bacterial growth, impairing the bacterial cell wall and indirectly supporting the antioxidant status of animals [23,24]. In addition, the inclusion of leonardite in the feed, characterised by a large amount of humic substances, likely stabilizes the microbial intestinal population, improving intestinal barrier health and preventing diarrhoea. Although the effect of leonardite, is still not fully understood, it could be due to the affinity of humic substances to biological membranes and their participation in ion transportation, which may boost performance and health status [27,28]. 

Humic acids contained in leonardite have shown an ability to lower pH in the gastrointestinal tract and stabilize intestinal flora [53]. Tributyrin also reduces diarrhoea due to its nutrient absorption, and the intestinal morphology of villi enhancement in weaned piglets [54]. Tributyrin and butyric acid lower the pH of GIT (particularly in the stomach and intestine) promoting an increase in beneficial bacteria [32]. 

The decrease in diarrhoea incidence and the positive effect on faecal consistency could be related to the in-feed supplementation of the mixture of functional compounds. These results are in line with other results showing a reduction in diarrhoea related to the inclusion of tannin extracts, leonardite and tributyrin individually supplemented in animal diets, and proposed as a valuable alternative to antibiotics for weaned piglets [28,55,56,57]. Intestinal integrity supported by tributyrin has been widely demonstrated in the literature, due to the release of three butyric acid molecules in the gut. The effects of lowering the pH and strengthening epithelium integrity could increase animal resistance to diarrhoea [33,57]. Each additive in the mixture is characterised by a different composition and mechanism of action. Hence, our results suggest that they could improve intestinal health through a dynamic interaction with the GIT environment.

Although numerous feed additives have been proposed in pig diets, there are contrasting results in the literature. Establishing one additive as an alternative to antibiotics in the feed is therefore not possible; however, since no antibiotics are used as growth promoters, some functional compounds will be beneficial when fed to pigs [58].

### 3.4. Serum Metabolic Profile 

The results from the serum metabolic profile revealed biochemical levels in line with reference values and with previously obtained data from single additive supplementation in both groups (Table 2) [20,27,30,59,60,61], thus confirming the absence of toxic effects on the main serum metabolic parameters. However, a significant reduction in LDL cholesterol was observed in the MIX group compared to the CTRL group (1.21 ± 0.08 and 1.48 ± 0.07, respectively; *p* < 0.05). Low-density lipoproteins are well known for their risk factors related to the development of circulatory diseases. Blood concentrations of LDL promote atherosclerosis and cardiovascular diseases [62] and low LDL serum levels are key for cardiovascular prevention and treatment [63]. 

Tributyrin can modulate lipid metabolism since short chain fatty acids can lower LDL cholesterol levels [64]. These particular forms of three esterified fatty acids have been associated with a low phosphorylated c-JUN-NH2 terminal kinase content with partial hepatic steatosis reversion, leading to a reduction in fat accumulation [65]. In our study, the decrease in LDL cholesterol level confirmed the encouraging results on the lipid metabolism modulation of leonardite and tributyrin supplementation, positively affecting the animal health status [27,30].

The MIX group revealed a statistically significant lower total protein content in blood serum compared to CTRL (47.75 ± 1.67 and 54.92 ± 1.46 g/L, respectively; *p* < 0.05). Tannins are able to establish stable and insoluble complexes with dietary proteins [66,67], which could slightly reduce the protein bioaccessibility in the gut, leading to a decrease in serum total protein. Although tannins could potentially reduce protein digestibility, we found no side effects related to animal performance, thus suggesting that the reduction did not impair animal performance and health, in line with Caprarulo et al. [20]. The blood serum urea level revealed a tendency to decrease in the MIX compared to the CTRL group (0.78 ± 0.07 and 0.99 ± 0.06, respectively; *p* < 0.09). Serum urea is a nonprotein nitrogen directly associated with CP concentration in the feed. Circulating urea could be considered a useful indicator for diet formulation and nitrogen use [68]. Caprarulo et al. [20] highlighted a shift in protein metabolism due to tannin supplementation after 40 days, leading to an increased bacterial protein synthesis. 

### 3.5. Microbiological Analysis of Faecal Samples

**Figure 2:**
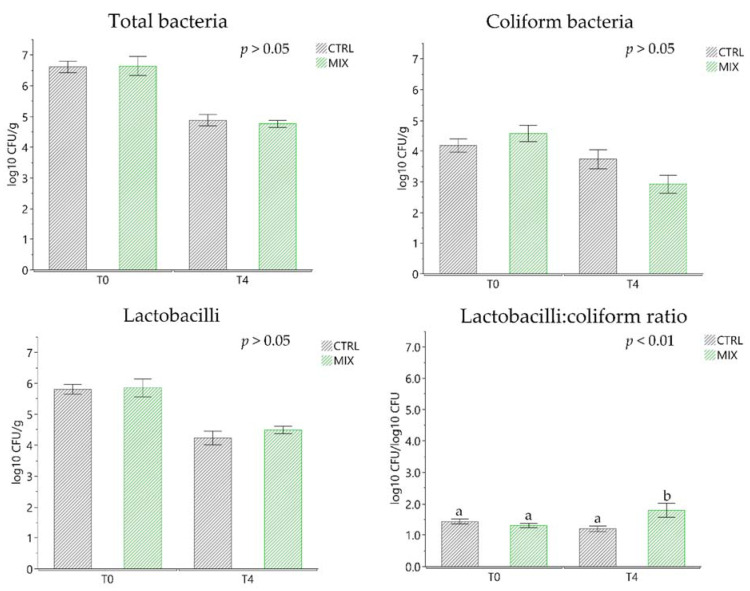
Faecal content of the principal bacterial groups (total bacteria, coliform, lactobacilli and lactobacilli:coliform ratio) divided by control (CTRL) and treatment (MIX) at the beginning (T0) and after 28 days of trial (T4). ^a,b^ Means with different superscripts are significantly different between treatments (*p* < 0.05). Data are expressed as least square means (LSMEANS) and standard errors (SE). CTRL: control group; MIX: treatment group supplemented with 0.75% tannin extract, 0.25% leonardite and 0.20% tributyrin in the diet.

Total plate counting, coliform and lactobacilli faecal content revealed no differences between groups at T0 and T4, highlighting a similar content in total bacteria and in coliform and lactobacilli in piglets’ faeces (Figure 2). T0 was characterised by a high prevalence of lactobacilli (5.81 ± 0.20 log_10_ CFU/g for CTRL and 5.85 ± 0.21 log_10_ CFU/g for MIX). On the other hand, T4 showed a statistically significant reduction of this group of bacteria (4.23 ± 0.21 log_10_ CFU/g for CTRL and 4.49 ± 0.21 log_10_ CFU/g for MIX; *p* < 0.0001). 

Before weaning, lactobacilli or lactic acid bacteria are usually higher in piglets due to the milk consumption, and may decrease naturally after weaning and feeding with solid diets [69]. In our study, the lactobacilli:coliform bacteria ratio increased at day 28 (T4) in the MIX compared to CTRL group (1.79 ± 0.13 and 1.20 ± 0.13 log_10_ CFU/log_10_ CFU, respectively; *p* < 0.01). The lactobacilli:coliform ratio predicts intestinal health and is used in efficacy tests of feed additives and acidifiers in order to promote immune defence. After weaning, the ratio changes depending on the level of coliform and immune defence development by the host. The lactobacilli:enterobacteria ratio is adopted as a simple index whose increase is related to a higher resistance to intestinal disorders [70]. 

### 3.6. Nitrogen Content, Apparent Nitrogen Digestibility, Volatile Fatty Acids and pH of Faecal Samples

The faecal nitrogen content revealed a statistically significant increase in the MIX compared to the CTRL group (4.19 ± 0.10 and 3.85 ± 0.10% on dry matter basis, respectively; *p* < 0.05) at T4. A linear positive correlation (r = 0.79; *p* < 0.0001) was observed for the faecal nitrogen content (fresh weight basis) and dry matter of faeces, highlighting that more solid faeces had a higher nitrogen concentration. The effects of tannins on decreasing protein bioavailability and forming indigestible complexes are well known [71]. The results suggest that the tannin extract supplemented in the MIX group diet increased the nitrogen excretion. However, this effect did not influence the animal growth performance, as confirmed by a non-statistically significant difference in the nitrogen excretion between the two experimental groups, considering nitrogen percentages on a fresh weight basis (1.13 ± 0.05% for CTRL and 1.24 ± 0.05% for MIX group). 

Although an increased nitrogen output suggests a lower protein availability, which is key for piglet growth, the performance showed no differences in pig growth. This was confirmed by the apparent nitrogen digestibility which revealed no statistically significant difference between the CTRL (80.96 ± 7.37%) and MIX (83.12 ± 6.75%) group. The availability of the nitrogen in the diet was thus not affected by the dietary treatment. These results are in line with Caprarulo et al. [20] who, after supplementing tannin extract in the feed, observed a shift in protein metabolism, which immediately promoted bacterial growth in the large intestine, which were able to exploit undigested substrate. No significant differences in faecal VFA content were detected between the two experimental groups (Table S3), suggesting that the microbial fermentations of the MIX group were not influenced by the treatment. 

The faecal pH measured at T0 and at T4 showed no statistically significant differences between the CTRL and MIX (7.00 ± 0.08 and 7.01 ± 0.08, respectively) groups, thus highlighting that the inclusion of the experimental mix in the animals’ diet did not influence this parameter. Faecal pH is a cheap method that provides important information on intestinal health. Faecal pH values over 8.5 could indicate ammonia formation from intestinal fermentation caused by decreased protein digestion [72].

The results suggest that neither leonardite (rich in organic acids) tannins (protein-binding ability) nor tributyrin (intestinal pH lowering effect) significantly impaired protein digestion, intestinal fermentation, and faecal pH. 

### 3.7. Microbiota Composition and Community Diversity Associated with Mix Supplementation

**Figure 3:**
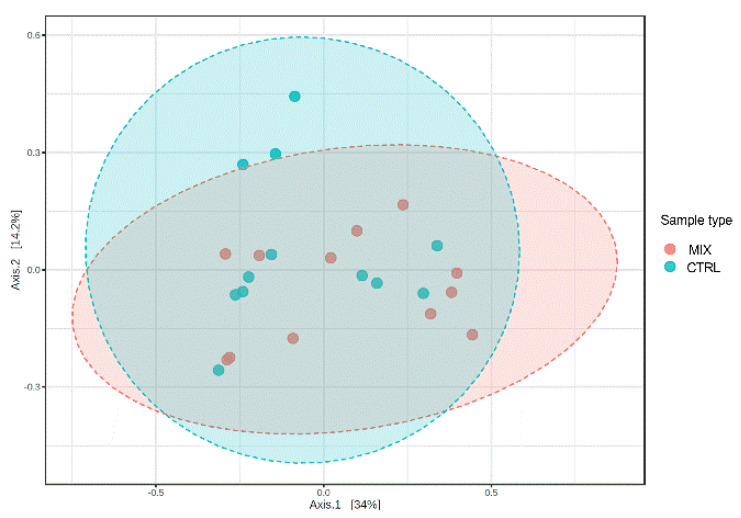
Principal coordinate analysis (PCoA, Bray–Curtis distance) plot of the gut microbiota of weaned piglets fed a diet with (MIX) or without mix supplementation (CTRL). CTRL: control group; MIX: treatment group supplemented with 0.75% tannin extract, 0.25% leonardite and 0.20% tributyrin in the diet.

A total of 264,055 filtered sequences were obtained in the samples collected from the MIX and CTRL groups. The median sequencing coverage was 52,668 sequences per sample. The beta-diversity was evaluated based on the PERMANOVA analysis of the Bray–Curtis distances. Samples were not clustered separately, although PERMANOVA analysis indicated significant differences between the two dietetic groups (R2 = 0.087, *p* < 0.038) (Figure 3). In addition, the MIX supplementation had no significant influence on the faecal bacterial alpha diversity calculated by Chao 1 (*p* = 0.29), observed OTUs (*p* = 0.55), Simpson (*p* = 0.86) and Shannon (*p* = 0.93) indices. As a consequence, no significant differences in species diversity and richness were observed among dietary groups. 

At the phylum level, the faecal microbiota collected from the control group were dominated by *Firmicutes* (89%) followed by *Bacteroidetes* (7.5%), whereas in animals receiving the MIX supplementation, *Firmicutes* represented 83% and *Bacteroidetes* 14%. The presence of *Actinobacteria* phylum was less than 1% in both groups. These differences between the two groups were significant for both *Firmicutes* (FDR = 0.023) and *Bacteroidetes* (FDR < 0.0001). The decrease in *Actinobacteria* was also significant (FDR = 0.023) in the treated group of animals. Among the less abundant phyla in the MIX group, *Fibrobacteres* was significantly higher (FDR < 0.001), whereas *Chlamydiae* and *Cyanobacteria* were significantly lower than the control group (Table 3). 

At the family level, *Prevotellaceae* and *Fibrobacteraceae* increased significantly in samples collected from the MIX group, whereas *Chlamydiaceae* decreased (*p* < 0.01; Table 4). Additionally, at the family level, weak correlations were detected. The *Fibrobacteriaceae* and *Prevotellaceae* families correlated positively with ADFI (r = 0.480, *p* = 0.018; and r = 0.563, *p* = 0.004, respectively) and FCR (r = 0.477, *p* = 0.018; r = 0.561, *p* = 0.004, respectively). 

At the genus level, a significant increase in *Prevotella* and *Fibrobacter* was detected in the piglets’ supplemented diet, whereas the relative abundance of RFN20, *Eubacterium*, *Lachnospira*, *Desulfovibrio* and *Chlamydia* was low (*p* < 0.01). The values of fold changes and FDR are reported in Table 4. The FCR of *Fibrobacter* (r = 0.477, *p* = 0.018) and *Prevotella* (r = 0.573, *p* = 0.03) showed very similar correlation values to those obtained with ADFI. *Fibrobacter* and *Prevotella* are well-known dietary fibres that degrade bacteria and produce short chain fatty acids [73]. 

The genus *Chlamydia* is a well-known cause of disease. Within the genus, several species have been identified and in particular, in pigs, *Chlamydia suis* seems to be widespread and related to the presence of other pathogens [74]. In weaned piglets, *Chlamydia* spp. has been associated with intestinal microscopic lesions in healthy [75] as well as in diarrhoeic animals [76].

**Figure 4:**
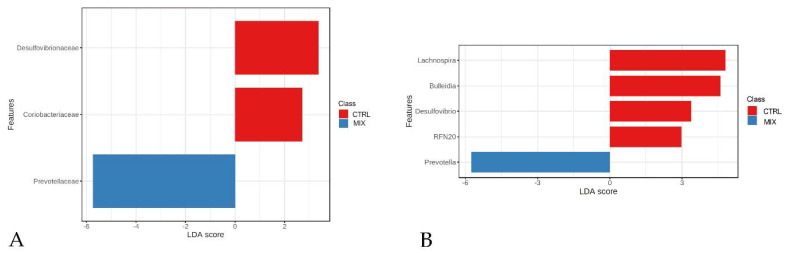
LEfSe analysis results between control (CTRL) and mix group (MIX) of animals at the family (**A**) and the genus level (**B**). CTRL: control group; MIX: treatment group supplemented with 0.75% tannin extract, 0.25% leonardite and 0.20% tributyrin in the diet.

LEfSe analysis revealed that the two dietary groups could be differentiated at the family level by *Desulfovibrionaceae*, *Coriobacteriaceae* and *Prevotellaceae* (Figure 4A). The first two families were more abundant in the CTRL group, whereas *Coriobacteriaceae* family was more abundant in the MIX group. 

**Figure 5:**
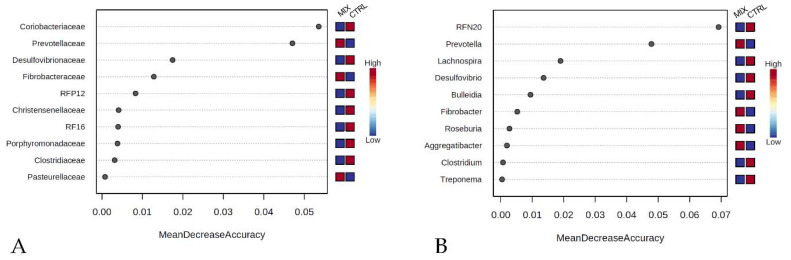
Random forest analysis results between mix fed animals (MIX) and control group (CTRL) at the family (**A**) and at the genus level (**B**) CTRL: control group; MIX: treatment group supplemented with 0.75% tannin extract, 0.25% leonardite and 0.20% tributyrin in the diet.

At the genus level, *Lachnospira*, RFN20, *Desulfovibrio* and *Bulleidia* characterised the gut microbiota of the CTRL group, whereas *Prevotella* showed a very high LDA score associated with MIX group samples (Figure 4B). These results were confirmed by the random forest analysis which indicated *Desulfovibrionaceae*, *Coriobacteriaceae* and *Prevotellaceae* families as differentiating the two dietary groups. The discriminant genera resulting from the previous analysis were in agreement with those of the random forest analysis (Figure 5A,B).

All these analyses thus confirmed that the MIX supplementation reduced *Desulfovibrionaceae* and *Coriobacteriaceae,* whereas it increased the abundance of *Prevotellaceae*. In the gut, *Coriobacteriaceae* have been associated with bile salts and steroid conversion. In addition, they have shown particular characteristics involved in the conversion of food polyphenols [77]. In humans, the family *Desulfovibrionaceae* and in particular the *Desulfovibrio* genus, have been associated with the inflammation status, as well as being involved in the disruption of the intestinal barrier [78]. In piglets, *Desulfovibrionaceae* increase during the weaning period and play a crucial role in H_2_ balance, thus maintaining suitable conditions for intestinal fermentation [79].

Concerning *Prevotellaceae*, contradictory results have been reported, in particular regarding their impact on pig performance. Negative correlations between animal body weight and abundance of *Prevotellaceae* have been described [80], while the *Prevotella*-dominant enterotype has been associated with higher feed intake values compared to the *Treponema*-dominant enterotype in Duroc pigs [81]. As suggested by Amat et al. [82], further analyses are required to clarify the role of specific species of *Prevotella* in pig performance. Within this family, *Prevotella copri* is the most abundant species found in pig gut microbiota after weaning. This species is present during the nursing period, increases at weaning, remains very abundant in the growth phases and decreases in the finishing phase [83]. The link between *Prevotella* species and the development of diarrhoea is still controversial. In fact, in some conditions, its high abundance has been associated with a preventive effect on diarrhoea development [84], while in other studies, the high presence of *Prevotella* spp. has been posited as promoting this disease [81]. Based on our results, the increased level of the *Prevotella* genus was not associated with a high faecal score or with the frequency of diarrhoea episodes. 

Finally, genera co-occurrence network analysis revealed relationships between *Prevotella* and *Lachnospira*, RFN20, *Desulfovibrio*, *Bacteroides*, *Eubacterium* and *Bulleidia*. The estimation of sparse correlations revealed a negative correlation between *Prevotella* and *Lachnospira* (SparCC = −0.67; *p* < 0.01), RFN20 (SparCC = −0.92; *p* < 0.01), *Desulfovibrio* (SparCC = −0.54; *p* = 0.03) and *Bulleidia* (SparCC = −0.62; *p* = 0.03) genera. These results indicate an antagonistic relationship between *Prevotella*, which was increased in the MIX-diet group, and bacterial genera which were, on the contrary, reduced due to the dietary treatment. Quebracho and Chestnut tannin extracts, leonardite and tributyrin supplementation seemed mainly to increase the genus *Prevotella*, which seems to modulate other microorganisms. Although the role of *Prevotella* has not yet been completely defined, in our study its presence was correlated with ADFI and not with diarrhoea. Further studies are needed to clarify the effects on the gut microbiota of this microbial genus.

## 4. Conclusions

We found that the dietary administration of a combination of Chestnut and Quebracho tannin extracts, leonardite and tributyrin to significantly reduce the occurrence of diarrhoea and increase the lactobacilli:coliform ratio after 28 days, thus promoting animal health. Functional compound supplementation also revealed the positive regulation of lipid metabolism, thus confirming the possible role of tributyrin and leonardite in modulating the fatty acid profile in blood serum. Our results indicated that this supplementation promotes changes to gut microbial communities, particularly increasing *Prevotella* spp. In conclusion, the in-feed supplementation of Quebracho and Chestnut tannin extracts, leonardite and tributyrin could be a promising alternative for the judicious use of antimicrobials in weaned piglets, which is considered a global sustainability priority. However, further studies are needed to better clarify the exact mechanism of action and the optimal concentration of these three functional compounds to maximise their effect on animal health and performance. 

## Figures and Tables

**Table 1:** Diet composition and principal chemical characteristics of in vivo trial (% as fed basis) divided by control (CTRL, fed basal diet) and treatment group (MIX, fed basal diet supplemented with 0.75% Quebracho and Chestnut Tannin extract, 0.25% leonardite and 0.20% tributyrin).

Ingredients, % as Fed Basis	CTRL	MIX
Barley, meal	26.84	26.84
Wheat, meal	12.45	10.45
Corn, flakes	11.63	11.63
Corn, meal	10.00	10.00
Barley, flakes	7.50	7.50
Soy protein concentrates	5.00	5.00
Biscuits, meal	4.00	4.00
Soybean, meal (44%)	4.00	4.00
Dextrose monohydrate	3.50	3.50
Sweet milk whey	2.50	2.50
Herring, meal	2.00	2.00
Plasma, meal	2.00	2.00
Beet pulp	1.40	1.40
Acidifiers ^1^	1.70	1.70
Coconut oil	1.00	1.00
Soy oil	1.00	1.00
Dicalcium phosphate	0.60	0.60
L-Lysine	0.60	0.60
Benzoic acid	0.50	0.50
Vitamins and mineral premix ^2^	0.50	0.50
DL-Methionine	0.39	0.39
L-Threonine	0.35	0.35
Sodium Chloride	0.27	0.27
L-Valine (96.5%)	0.12	0.12
Enzyme mix ^3^	0.10	0.10
L-Tryptophan	0.05	0.05
Experimental mix ^4^	-	2.00
**Calculated Chemical Composition ^5^**		
Crude protein (%)	18.65	18.33
Fat (%)	4.78	4.75
Crude fibre (%)	3.00	2.98
Ashes (%)	5.52	5.48
DE ^6^ (Mc/Kg)	3.92	3.83

^1^ Citric acid, fumaric acid, orthophosphoric acid, sorbic acid, calcium formate. ^2^ Additives per Kg: Vitamins, pro-vitamins and substances with similar effect. Retinyl Acetate 15000 IU, Vitamin D3-Cholecalciferol 2000 IU, Vitamin E 120 mg, Vitamin B1 2.0 mg, Vitamin B2 4.8 mg, Vitamin B6 3.4 mg, Calcium D-pantothenate 15.0 mg, Vitamin B12 0.030 mg, Vitamin K3 1.9 mg, Biotin 0.19 mg, Niacinamide 30.0 mg, Folic Acid 0.96 mg, Vitamin C 144 mg, Choline chloride 288 mg, Betaine hydrochloride 1000 mg, Compounds of trace elements Iron sulphate 115 mg, Manganese Oxide 48.0 mg, Zinc Oxide 96.1 mg, Copper Oxide 130 mg, Anhydrous Calcium Iodate 0.96 mg, Sodium Selenite 0.34 mg. ^3^ 6-phytase, endo-1,4-beta-xylanase, endo-1,3(4)-beta-glucanase. ^4^ The experimental mix was composed of 0.80% wheat flour 00 and the three supplemented additives: 0.75% Quebracho and Chestnut tannin extracts (Silvafeed^®^ Nutri P, Silvateam, Mondovì, Italy), 0.25% leonardite (New Feed Team, Lodi, Italy), 0.20% tributyrin (ACIFIS^®^ Tri-B, New Feed Team, Lodi, Italy). ^5^ Calculation performed with Purimix System^®^ software (Fabermatica, Cremona, Italy). ^6^ DE: digestible energy content estimated from NRC (2012).

**Table 2:** Metabolic profile of blood serum divided by control (CTRL) and treatment group (MIX) measured at day 28.

Serum Metabolite	CTRL	MIX	SE CTRL	SE MIX	*p*-Value
Total protein content, g/L	54.92 ^a^	47.75 ^b^	1.46	1.67	0.0258
Albumin, g/L	27.89	25.91	1.24	1.42	0.3808
Globulin, g/L	25.85	23.31	1.44	1.62	0.2938
Albumin/Globulin (A/G)	1.06	1.18	0.06	0.07	0.2465
Urea, mmol/L	0.99	0.78	0.06	0.07	0.0746
Alanine aminotransferase (ALT-GPT), IU/L	54.75	56.81	4.68	5.27	0.7867
Total bilirubin, µmol/L	1.90	1.80	0.11	0.13	0.5750
Glucose, mmol/L	6.19	6.91	0.48	0.55	0.4169
Phosphorus, mmol/L	3.26	3.10	0.13	0.15	0.4547
Magnesium, mmol/L	0.92	0.88	0.04	0.05	0.6328
Creatinine, µmol/L	70.18	65.27	3.81	4.47	0.5098
Total cholesterol, mmol/L	2.62	2.36	0.12	0.13	0.1901
High density lipoprotein (HDL), mmol/L	1.01	1.04	0.07	0.08	0.7955
Low density lipoprotein (LDL), mmol/L	1.48 ^a^	1.21 ^b^	0.07	0.08	0.0435
Triglycerides, mmol/L	0.60	0.69	0.09	0.10	0.4982
Insulin, mU/L	10.78	9.47	2.57	2.87	0.7437
Glucagon, pg/mL	294.10	286.51	13.99	22.38	0.8103

^a,b^ Means with different superscripts are significantly different between treatments (*p* < 0.05). Data are expressed as least square means (LSMEANS) and standard errors (SE). CTRL: control group; MIX: treatment group supplemented with 0.75% tannin extracts, 0.25% leonardite and 0.20% tributyrin in the diet.

**Table 3:** Differentially abundant phyla between MIX and CTRL groups of piglets.

Phylum	log2FC	Log CPM	*p*-Values	FDR
*Bacteroidetes*	1.973	17.166	<0.0001	<0.0001
*Fibrobacteres*	2.736	11.433	<0.0001	0.0002
*Chlamydiae*	−2.860	10.731	0.0058	0.0192
*Actinobacteria*	−1.449	6.976	0.0101	0.0238
*Firmicutes*	1.119	20.203	0.0119	0.0238
*Cyanobacteria*	−1.566	7.227	0.0274	0.0456
*Verrucomicrobia*	−2.050	11.582	0.0356	0.0509

CTRL: control group; MIX: treatment group supplemented with 0.75% tannin extract, 0.25% leonardite and 0.20% tributyrin in the diet.

**Table 4:** Differentially abundant families and genera between MIX fed animals and CTRL group.

Family	log2FC	Log CPM	*p*-Values	FDR
*Prevotellaceae*	2.010	16.721	<0.0001	<0.0001
*Fibrobacteraceae*	2.359	11.288	0.0004	0.0053
*Chlamydiaceae*	−3.149	10.902	0.0035	0.0326
**Genus**				
*Prevotella*	1.963	16.848	<0.0001	0.0003
RFN20	−2.295	7.274	<0.0001	0.0003
*Eubacterium*	−4.652	14.923	<0.0001	0.0003
*Fibrobacter*	2.396	11.328	0.0006	0.0047
*Lachnospira*	−2.050	12.780	0.0032	0.0214
*Desulfovibrio*	−2.301	8.221	0.0081	0.0443
*Chlamydia*	−2.701	10.827	0.0107	0.0503

CTRL: control group; MIX: treatment group supplemented with 0.75% tannin extract, 0.25% leonardite and 0.20% tributyrin in the diet.

## Data Availability

The data presented in this study are available within the article and Supplementary Materials.

## Supplementary Materials

The following are available online at https://www.mdpi.com/article/10.3390/ani11061693/s1, Table S1: Chemical composition of experimental diets: CTRL (basal diet) and MIX (basal diet supplemented with 0.75% of Quebracho and Chestnut tannin extracts, 0.25% of leonardite, 0.20% of tributyrin), Table S2: Zootechnical performance of the experimental trial (from day 0 to 28) divided by control (CTRL) and treatment (MIX) group, Table S3: Mean values of faecal VFA proportion of tannin extracts, leonardite and tributyrin supplementation (MIX) and control (CTRL) groups.
