# Supplementary material for: Evaluation of Tannin Extracts, Leonardite and Tributyrin Supplementation on Diarrhoea Incidence and Gut Microbiota of Weaned Piglets
Source: Animals (Basel). 2021 Jun 6;11(6):1693. doi: 10.3390/ani11061693 (PMC8229630; doi:10.3390/ani11061693)
Supplement: Supplementary file 1 [file animals-11-01693-s001.zip › animals-1201580-supplementary.pdf]

Supplementary materials

Supplementary Table 1. Chemical composition of experimental diets: CTRL (basal diet) and MIX (basal diet supplemented with 0.75% of Quebracho and Chestnut tannin extracts, 0.25% of leonardite, 0.20% of tributyrin).

Analyte	CTRL	MIX
DM	90.89	91.01
CP	18.82	18.43
EE	4.38	4.58
CF	3.96	3.58
Ashes	5.05	4.93

DM: dry matter; CP: crude protein; EE: ether extract; CF: crude fiber.
All values are expressed as percentage as fed basis (%).
CTRL: control group; MIX: treatment group.

Supplementary Table 2. Zootechnical performance of the experimental trial (from day 0 to 28) divided by control (CTRL) and treatment (MIX) group.

				P-values		
	**CTRL**	**MIX**	**SEM±**	Trt	Time	Trt x Time
**BW, kg**				0.8789	< 0.0001	0.4992
d 0	7.46	7.50	0.20			
d 7	8.34	8.27	0.20			
d 14	10.05	9.97	0.20			
d 21	12.30	12.43	0.20			
d 28	14.10	14.28	0.20			
**ADFI, kg/d**				0.1966	< 0.0001	0.2433
d 0-7	0.268	0.202	0.03			
d 7-14	0.490	0.526	0.03			
d 14 -21	0.566	0.519	0.03			
d 21-28	0.656	0.575	0.03			
**ADG, kg/d**				0.4787	< 0.0001	0.3804
d 0-7	0.096	0.088	0.01			
d 7-14	0.244	0.243	0.01			
d 14 -21	0.320	0.353	0.01			
d 21-28	0.363	0.368	0.01			
**FCR, kg/kg**				0.6895	0.0003	0.7659
d 0-7	2.76	2.80	0.25			
d 7-14	2.00	2.19	0.25			
d 14 -21	1.77	1.51	0.25			
d 21-28	1.84	1.59	0.25			

Data are expressed as least square means (LSMEANS) and standard error of the means (SEM).
BW: body weight; ADG: average daily gain; ADFI: average daily feed intake; FRC: feed conversion rate; CTRL: control group; MIX: treatment group supplemented with 0.75% tannin extract, 0.25% leonardite and 0.20% tributyrin in the diet.

Supplementary Table 3. Mean values of faecal VFA proportion of tannin extracts, leonardite and tributyrin supplementation (MIX) and control (CTRL) groups.

	**Acetate mmol % ± SD**	**Propionate mmol % ± SD**	**Isobutyrate mmol % ± SD**	**Butyrate mmol % ± SD**	**Isovalerate mmol % ± SD**	**Valerate mmol % ± SD**
CTRL	56.38±5.02	21.69±1.55	2.08±0.75	13.13±2.65	3.23±1.33	2.85±0.93
MIX	59.07±3.71	21.38±1.26	1.92±0.56	11.43±3.05	2.90±0.94	2.76±0.76
p-value	0.1487	0.5962	0.5598	0.1591	0.4901	0.5892

CTRL: control group; MIX: treatment group supplemented with 0.75% tannin extract, 0.25% leonardite and 0.20% tributyrin in the diet; VFA: volatile fatty acids.
